# Unscrambling cancer genomes via integrated analysis of structural variation and copy number

**DOI:** 10.1016/j.xgen.2022.100112

**Published:** 2022-03-22

**Authors:** Charles Shale, Daniel L. Cameron, Jonathan Baber, Marie Wong, Mark J. Cowley, Anthony T. Papenfuss, Edwin Cuppen, Peter Priestley

**Affiliations:** 1 Hartwig Medical Foundation Australia, Sydney, NSW, Australia; 2 Hartwig Medical Foundation, Science Park 408, Amsterdam, the Netherlands; 3 Bioinformatics Division, Walter and Eliza Hall Institute of Medical Research, Parkville, VIC, Australia; 4 Department of Medical Biology, University of Melbourne, Melbourne, VIC, Australia; 5 Children’s Cancer Institute, Lowy Cancer Centre, UNSW Sydney, Kensington, NSW, Australia; 6 School of Women’s and Children’s Health, UNSW Sydney, Kensington, NSW, Australia; 7 Peter MacCallum Cancer Centre, Melbourne, VIC, Australia; 8 Sir Peter MacCallum Department of Oncology, University of Melbourne, Melbourne, VIC, Australia; 9 Center for Molecular Medicine and Oncode Institute, University Medical Center Utrecht, Heidelberglaan 100, Utrecht, the Netherlands

**Keywords:** cancer genomics, structural variation, genomic rearrangement, LINX, homozygous disruption, gene fusion, mobile element insertion, ecDNA, genomic shard, reciprocal duplication

## Abstract

Complex somatic genomic rearrangements and copy number alterations are hallmarks of nearly all cancers. We have developed an algorithm, LINX, to aid interpretation of structural variant and copy number data derived from short-read, whole-genome sequencing. LINX classifies raw structural variant calls into distinct events and predicts their effect on the local structure of the derivative chromosome and the functional impact on affected genes. Visualizations facilitate further investigation of complex rearrangements. LINX allows insights into a diverse range of structural variation events and can reliably detect pathogenic rearrangements, including gene fusions, immunoglobulin enhancer rearrangements, intragenic deletions, and duplications. Uniquely, LINX also predicts chained fusions that we demonstrate account for 13% of clinically relevant oncogenic fusions. LINX also reports a class of inactivation events that we term homozygous disruptions that may be a driver mutation in up to 9% of tumors and may frequently affect *PTEN*, *TP53*, and *RB1*.

## Highlights

• LINX is an algorithm to classify somatic structural variation in tumors
• Chaining, clustering, and visualizations provide insights into complex rearrangements
• LINX predicts diverse pathogenic rearrangements, including chained fusions
• Homozygous disruptions are a distinct and common driver class in tumors

## Graphical abstract

**Figure:**
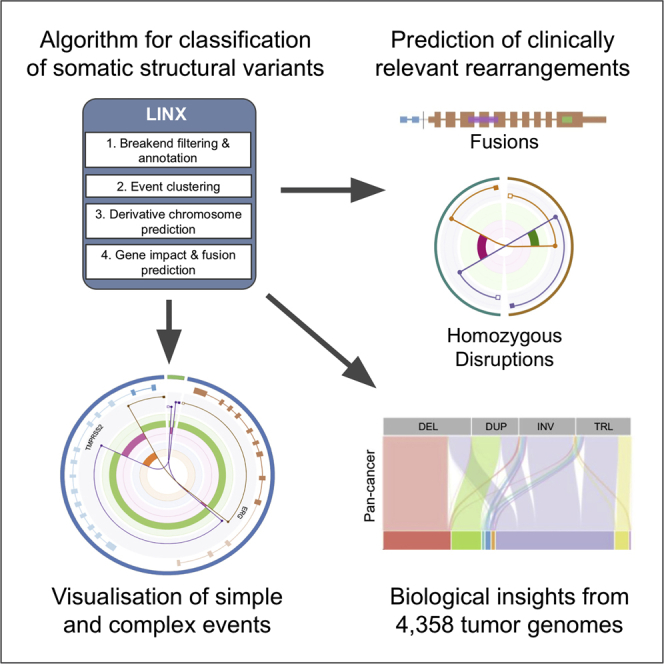

## Teaser

Shale et al. present a comprehensive analysis of somatic structural variation in a large cohort of cancer genomes. They have developed an algorithm, LINX, that reveals insights into complex genomic events and demonstrates the utility of whole-genome sequencing in detection of diverse clinically relevant gene fusions and disruptions.

## Introduction

Somatic structural variation (SV) and associated copy number alterations (CNAs) are key mechanisms in tumorigenesis.^1^ However, both the mechanisms driving and the consequences of genomic rearrangements in cancer are less well understood than for point mutation events. This is due both to the relative paucity of whole-genome sequencing (WGS) data that are required for comprehensive SV analysis and also to the fact that genomic rearrangements have significant diversity. Many rearrangements involve a high degree of complexity, with individual events resulting in multiple or even hundreds of breaks.^2^^,^^3^ Interpretation of these highly rearranged genomes is challenging but simultaneously highly relevant for the identification of driver events that may function as biomarkers or druggable targets.

LINX is an SV interpretation tool, which integrates CNA and SV calling derived from WGS data and comprehensively clusters, chains, and classifies genomic rearrangements. The motivation for this is twofold: first, from a biological perspective, to allow better insight into distinct mechanisms of rearrangements in tumorigenesis and second, from a clinical perspective, to allow prediction of the functional impact of structural rearrangements, including gene fusions and disruptions. A number of previous tools have been developed to analyze the roles of certain rearrangement event types in tumorigenesis, such as chromothripsis,^2^ chromoplexy,^4^ long interspersed nuclear element (LINE) insertions,^5^ and amplification mechanisms.^6^ Clustering methodologies have also been used previously to propose signatures of structural rearrangement.^1^^,^^7^ LINX goes further than just integrating the functionality of each of these previous tools, both by classifying all classes of rearrangements in each tumor genome and by predicting the local chained structure of the derivative chromosome as well as the functional impact of the rearrangement in a single application.

## Results

### LINX algorithm

The input for LINX is a base-pair-consistent segmented copy number and SV callset from the previously described tools PURPLE^8^ and GRIDSS.^9^ The base pair consistency means that each and every copy number change in the genome is matched precisely to an SV junction, which is represented either as a breakpoint when the partner location is known or as a single breakend when the partner location cannot be unambiguously determined.

**Figure 1:**
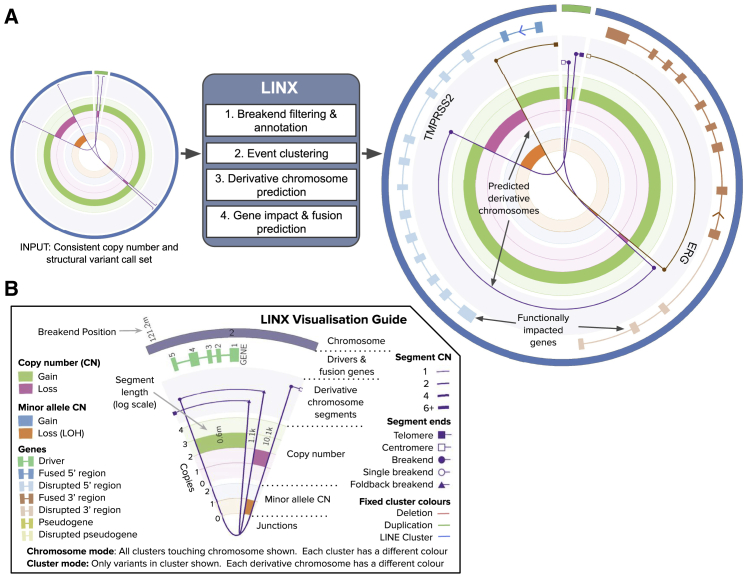
LINX schematic and visualizations (A) The LINX algorithm works in four steps to annotate, cluster, chain, and determine the functional impact of an integrated copy number. The Circos on the left represents the input of LINX and shows three structural variants (purple lines) affecting two chromosomes (outer track in green and blue) with consistent copy number breakpoints (middle track showing green for gain and red for loss). The Circos on the right shows example output of LINX, including the chaining of the variants into two continuous predicted derivative chromosomes (lines in brown and purple) and a canonical TMPRSS2_ERG fusion (genes depicted in blue and light brown on second outer circle with fused exons showing darker shading) on one of the two predicted chromosomes. (B) A detailed guide to the visualizations produced by LINX.

There are four key steps in the LINX algorithm (Figure 1; Methods S1). First, LINX annotates each breakpoint and breakend with several basic geometric and genomic properties that are important to the clustering and chaining algorithm. This includes whether each breakend is part of a foldback inversion, flanks a region of loss of heterozygosity (LOH), or is in a well-known fragile site region.^8^^,^^10^ LINX also annotates well-known line element source locations^5^ and identifies additional suspected mobile LINE source elements based on both the local breakpoint structure and signals of poly-A sequence insertions.

Second, LINX performs a clustering routine to group raw structural variants into distinct rearrangement “events.” LINX defines a rearrangement event as one or more junctions that likely occurred proximately in time and transformed the genome from one stable configuration to another. Events can range from a simple deletion or tandem duplication to complex events, including chromothripsis or breakage fusion bridge^11^ cascades. The fundamental principle for clustering in LINX is to join breakpoints where it is highly unlikely that they would have occurred independently. Rather than a single rule, such as clustering variants into events based solely on proximity^12^ or variants that form a “deletion bridge,”^4^ LINX employs a set of 11 independent rules in its clustering routine (Methods S1). These include clustering variants that are very close in proximity (<5 kb between breakends); clustering breakends that together delimit an LOH event, homozygous deletion, or region of high major allele copy number; clustering translocations that share common arms at both ends; clustering inversions, long deletion, and long tandem duplication variants that directly overlap each other; and clustering all foldback inversions that occur on the same chromosome arm.

Third, after resolving all variants into clusters, LINX predicts the derivative chromosome structure via a chaining algorithm. To do this, LINX considers all pairs of facing breakends on each chromosomal arm within each cluster and iteratively prioritizes which pair is most likely to be joined. The chaining logic imposes allele specific copy number constraints at all points on each chromosome and also the biological constraint that chromosomes are not permitted without a centromere unless strict criteria relating to detection of extrachromosomal DNA are met. Foldback inversions are also explicitly modeled to allow chaining of clusters of variable junction copy number and high amplification. Overall, the chaining prioritization scheme is designed to be error tolerant and aims to maximize the chance that each individual breakend is linked correctly to the next breakend on the derivative chromosome. However, due to multiple possible paths, upstream sources of error, and missing information, the prediction is representative only and, in the case of highly complex clusters, unlikely to be correct across all break junctions.

The fourth and final step in LINX is to annotate the gene impact of SV junctions to predict gene disruptions and fusions. Any breakend overlapping or in the upstream region of an Ensembl transcript^13^ is annotated with its position and orientation relative to the strand of the gene and the nearest splice acceptor or donor. Gene fusions are called by searching for correctly oriented splice acceptor and donor pairs on the predicted derivative chromosome, including chained fusions that may span multiple break junctions.^14^ To meet the fusion calling criteria, the breakends must also connect to viable contexts in each gene and not be terminated by further breakends in the chain on either 5′ or 3′ partner end (Methods S1). Since complex rearrangements may result in many candidate gene fusions, LINX streamlines clinical interpretation by providing a curated list of known pathogenic fusion gene pairs, as well as known promiscuous 5ʹ and 3ʹ fusion gene partners, and marks matching fusions as reportable. Finally, LINX also matches amplification, deletion, and LOH drivers called by PURPLE across a panel of well-known cancer genes (Table S1)^8^ to specific SV clusters and calls additional disruption driver events in tumor suppressor genes.

### Pan-cancer landscape of genomic rearrangements

**Figure 2:**
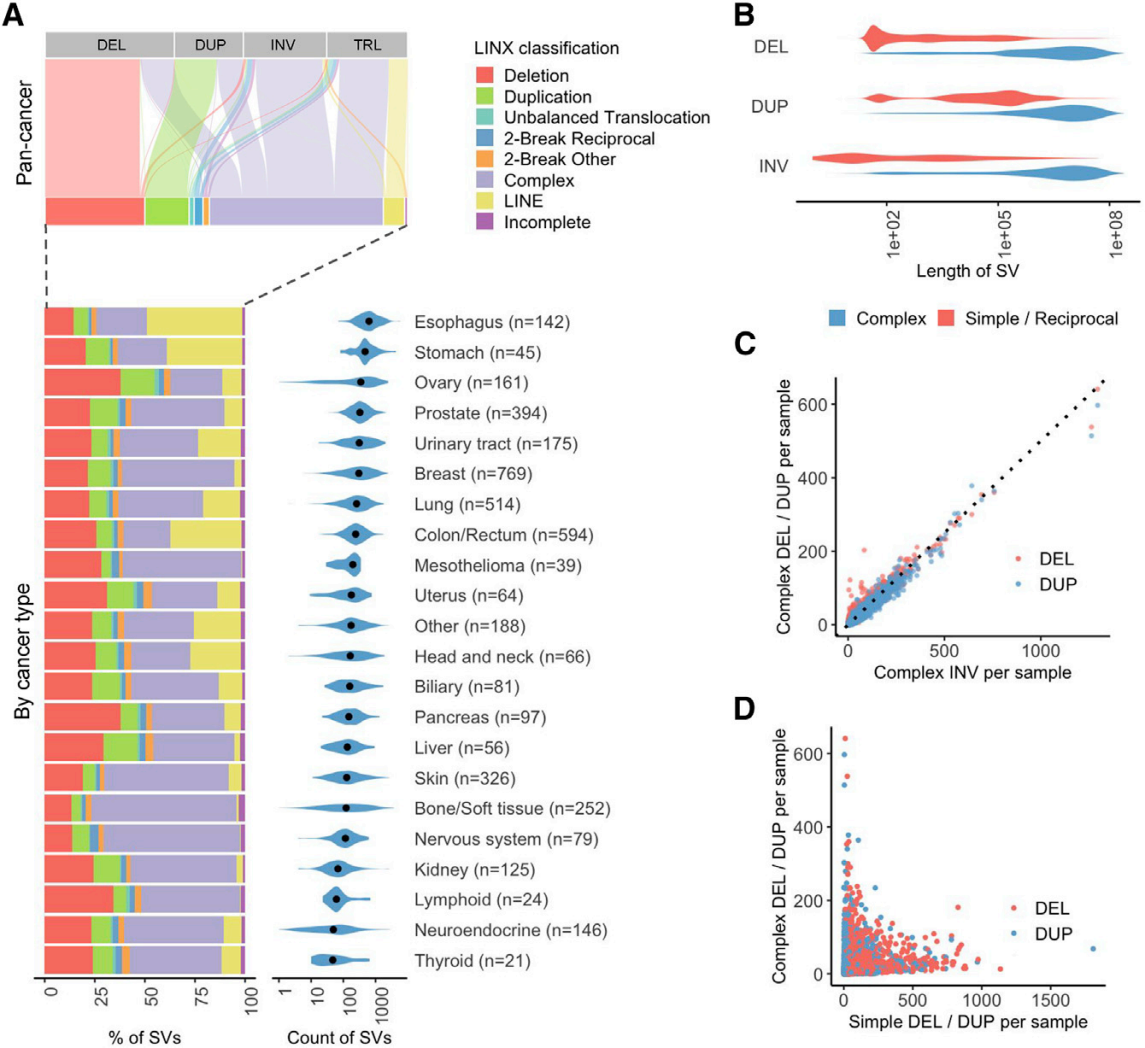
Landscape of genomic rearrangements (A) Top panel shows an alluvial plot depicting the proportional assignment of each of the raw structural variant types (DEL, deletion; DUP, duplication; INV, inversion; TRL, translocation) to LINX classification. The LINX classifications are further broken down by tumor type in a relative bar chart in the left lower panel. The right lower panel shows the distribution of the number of structural variants per sample grouped by tumor type, with the black dots indicating the median values. (B) Length distribution of notional deletion, duplications, and non-foldback inversions for both simple rearrangements and complex clusters (containing three or more variants). Note that foldback inversions have a distinct length distribution and are shown separately in Figure S4C. (C) Counts of deletions and duplications in complex clusters per sample both closely follow a 1:2 ratio (indicated by dotted line) compared with inversions, as expected by random rearrangements following catastrophic events. (D) Counts of simple deletions and duplications per sample are not correlated with counts of deletions and duplications in complex clusters. See also Figure S4 and Table S3.

To demonstrate the functionality of LINX, we ran it on a pan-cancer cohort of 4,358 paired tumor-normal, whole-genome-sequenced (median of 106× and 38× paired-end sequencing coverage, respectively) adult metastatic cancer samples from Hartwig Medical Foundation (referred to as Hartwig cohort; Table S2).^8^ Of these samples, 1,924 had matched whole transcriptome sequencing data, which were used for orthogonal validation where appropriate. Overall, we found a mean of 324 rearrangement junctions per sample with the highest rates in esophagus (mean = 753) and stomach (mean = 647) tumors and lowest rates in thyroid (mean = 102) and neuroendocrine (mean = 109) tumors (Figure 2A; Table S3). Event classification by LINX highlighted the diversity and tumor type specificity of rearrangement mechanisms with deletions, tandem duplications, LINE insertions, and complex events (defined as events with three or more junctions) found to be the largest classes of rearrangements in agreement with previous pan-cancer whole-genome analysis.^1^ We examined each of these event classifications in detail as follows.

#### Classification of simple and complex rearrangement events

Classification of event types in LINX can considerably simplify interpretation of a cancer genome. An important use case is to distinguish simple events driven by a single break resulting in deletions and duplications (Figure S1) from variants that are notionally called deletions and duplications by an SV caller but may be part of a more complex event. Clean mutational profiles for simple deletions and duplications are important for downstream applications, such as signature analysis^1^ and in particular homologous recombination (HR) deficiency classification,^15^^,^^16^ which is associated with both short deletions and tandem duplications and may be relevant to cancer treatment.

In the Hartwig cohort, we find that lengths of deletions and duplications classified as simple events are notably shorter than those clustered in complex events (Figure 2B). Moreover, the simple deletions and duplications show distinct characteristic length peaks, which have been previously shown to be associated with *BRCA1*, *BRCA2*, and *CDK12* inactivation or *CCNE1* amplification,^17^ as well as a short DUP signature that we have recently shown to be associated with colorectal tumors.^9^ On the other hand, the deletions and duplications involved in complex events have length distributions closely resembling that of inversions clustered in complex events. We also find that the per-sample counts of deletions, duplications, and inversions in complex events closely follows a 1:1:2 ratio as expected from random rearrangements following a catastrophic event (Figure 2C). However, the counts of simple deletion and duplication junctions per sample were only very weakly positively correlated with those for deletions or duplications that are categorized as part of complex events (deletions r = 0.156; duplications r = 0.13; Figure 2D). Taken together, these observations suggest LINX is able to accurately distinguish between simple and complex rearrangements.

LINX annotates every cluster involving two break junctions (further referred to as two-break junction events) with a resolved type where they can be consistently chained (Figure S2) or marks as “incomplete” where they cannot form a consistent set of derivative chromosomes (Figure S3). Consistent two-break junction clusters fall into two major categories—reciprocal events (e.g., reciprocal inversions or translocations) or events with insertions of a templated sequence either in a chain or cycle.^1^ We observe that two-break junction events with insertion sequences frequently involve very-short-templated sequences <1 kb in length, referred to as “genomic shards,”^18^ which we find to be pervasive in cancer, constituting 14% of somatic breakpoints. Genomic shards can confound classification of otherwise simple variant types, because a short-templated insertion from another chromosome appears notionally as two translocations and can easily be misinterpreted as a reciprocal translocation or more complex event.

LINX classifies events that can be resolved as a simple deletion, tandem duplication, or translocation event with one or more inserted shards as a “synthetic” event, under the assumption that the structure is likely created by the disruption of a simple event with the insertion of the templated sequence during repair without affecting the shard donor locus. In support of this hypothesis, we find that samples with high counts of simple deletion and duplications have significantly higher (p < 1 × 10^−60^ for both) counts of synthetic deletion and duplications, respectively (Figures S4A and S4B), and furthermore, we observe the lengths of synthetic deletions and duplications to be highly consistent with the respective lengths of simple deletions and duplications (Figure S4C). Synthetic deletion and duplication events can have many different breakend topological rearrangements, depending on the source and orientation of the inserted shard (Figure S1). Insertion of genomic shards is by no means unique to simple deletion and duplication events, as we also see frequent short-templated insertion sequences in breaks of more complex events, including foldback inversion and chromothripsis events. Synthetic foldback inversions also show the same length distribution as simple foldbacks (Figure S4C).

Reciprocal events are the other major category of two-break junction events. These arise from the crossover of multiple concurrent double-stranded breaks forming either a reciprocal inversion if both breaks occur on a single chromosome (with the segment in between the two breaks repaired inverted) or a reciprocal translocation if the repair is interchromosomal. Although reciprocal inversions and translocations are found in 65% of samples in the Hartwig cohort, they are infrequent relative to other events in cancer, making up 0.8% and 0.5% of all clusters, respectively. In addition to these classical reciprocal events, we also find other configurations of reciprocal events involving two break junctions (Figure S2). One prominent configuration that we term “reciprocal duplication” involves a pair of reciprocal translocations or inversions but with breakends facing each other at both ends with substantial overlap, often multiple kilobase or even megabase in length (Figure S4D). Reciprocal duplications are significantly enriched (p < 1 × 10^−60^) in samples with strong tandem duplication signatures (Figure S4E). Furthermore, the length distribution of reciprocal duplications matches the length distribution of the signature for samples with drivers known to cause tandem duplication phenotypes, i.e., *BRCA1*, *CCNE1*, or *CDK12* drivers (Figure S4F). This suggests that these reciprocal duplication events may arise from the same process that forms tandem duplication events, likely when multiple tandem duplications occur simultaneously in a cell and, instead of repairing locally, they may cross over and create a reciprocal duplication. This observation places constraints on the mechanism by which tandem duplications may form, because it requires duplication of DNA at both loci prior to breakage and is consistent with a replication restart-bypass model,^19^ but not a microhomology-mediated, break-induced replication model.^20^

#### Mobile element and pseudogene insertion detection

Somatic integration of LINEs is a common feature in many types of cancer, particularly esophagus and head and neck cancers.^5^ A LINE insertion may involve either the transposition of a full or partial LINE source element or the transduction of a partnered or orphaned genomic region within 5 kb downstream of the LINE element. While LINE insertions are typically simple events in themselves, correct classification of these break junctions is important to accurate interpretation of the genome, as they can otherwise be mistaken as translocations and other complex events.

LINE integrations can be difficult to resolve with short read technology, because the inserted sequence is often not uniquely mappable in the genome and typically includes a Poly-A tail,^21^ making assembly difficult. LINX circumvents both these issues by leveraging GRIDSS’s single breakend-calling capability^9^ to identify LINE insertion sites with breakend evidence for either repetitive LINE sequence, PolyA sequence, or a list of known recurrently active LINE source elements. To validate LINX’s detection of mobile element insertions, we ran LINX on 75 samples from the pan-cancer analysis of whole genomes (PCAWG) pan-cancer cohort and compared LINX’s LINE insertion calls with those from TraFiC-mem.^5^ Overall, 339 of 564 (60%) LINX LINE insertions calls were also detected by TraFiC-mem, with TraFiC-mem calling an additional 270 insertions not found by LINX. The concordance in total LINE insertion count was very strong on a per-sample basis (Figure S5A; Table S4), with most of the private calls in both pipelines being found in the high LINE mutational burden samples (Figure S5B), suggesting that many of the private calls from both pipelines may be genuine LINE insertions.

**Figure 3:**
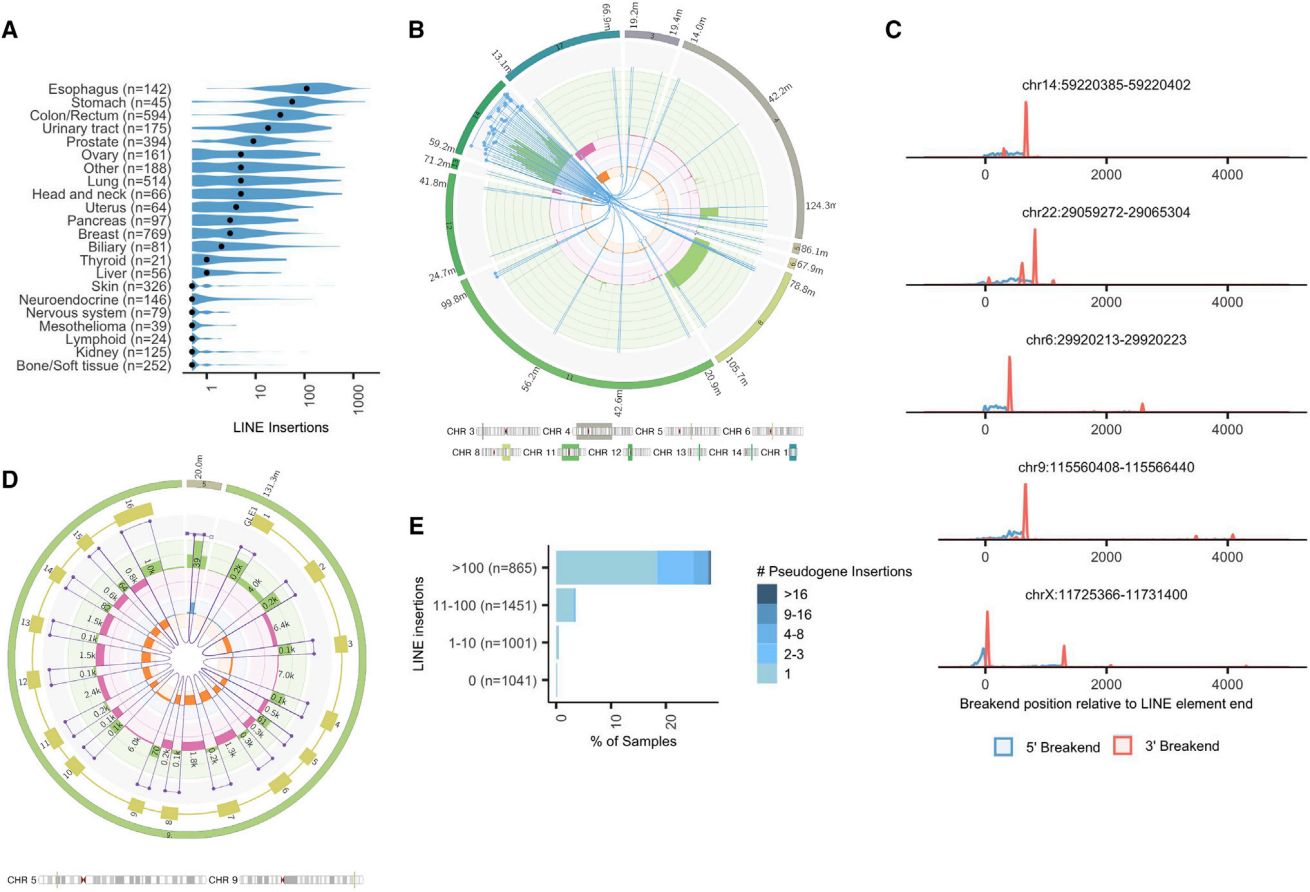
Mobile element insertions (A) Violin plot showing the distribution of the number of LINE insertions per sample grouped by tumor type. Black dots indicate the median values for each tumor type. (B) Complex LINE cluster in HMF002232B, a colorectal cancer. Overlapping segments from the LINE source element from chr14:59.2M have been inserted in at least 20 independent locations scattered throughout the genome. (C) Histogram showing frequency of breakends positions for all mobile element transductions in Hartwig cohort originating from the five most active LINE source elements relative to the last base of the LINE source element. (D) Pseudogene insertion of *GLE1* into an overlapping break junction on chromosome 5 in HMF002165A, a non-small cell lung cancer. All 16 exons of the *GLE1* canonical transcript are inserted, but parts of the first and last exons are lost. (E) Samples with high numbers of LINE insertions also have high numbers of pseudogene insertions. See also Figure S5 and Table S4.

Across the full Hartwig cohort, LINX found 76% of tumors have at least one LINE insertion event. Some tumors suffer extreme deregulation, with 6.7% of tumors having over 100 insertions and 2,241 insertions found in a single esophagus tumor sample (Figures 3A and 3B). The five most frequently inserted LINE source elements in the Hartwig cohort were all among the top six reported previously in the PCAWG pan-cancer cohort:^5^ chr22:29,059,272–29,065,304, chrX:11,725,366–11,731,400, chr14:59,220,385–59,220,402, chr9:115,560,408–115,566,440, and chr6:29,920,213–29,920,223. Analysis of the precise breakend locations at these five sites reveals highly recurrent site-specific patterns of transduction (Figures 3C and S5C), where the 3′ ends of the transduced sequences are normally sourced from a handful of specific downstream sites (presumably polyadenylation sequences of alternative transcription endpoints for the LINE source element), whereas the location of 5′ side of the transduction appears to be relatively randomly distributed.

At the LINE insertion site, accurate breakpoint determination can also give insight into potential biological mechanisms. LINX finds frequent target-site duplication^22^ but intriguingly finds two peaks in the distance between the insertion breakends, one at an overlap of 16 bases but also a second peak with no overlap, suggesting the possibility of two distinct breakage mechanisms for the second strand after LINE invasion (Figure S5D). Furthermore, for the 20% of insertions where LINX observed a 5′ inversion in the insertion sequence (due to twin priming),^22^ only a single peak with target site duplication of 16 bases is found.

LINX can also detect somatic pseudogene insertions resulting from the activated reverse transcriptase activity associated with deregulated LINE activity in tumors.^5^ LINX annotates any group of deletions that matches the exact boundaries of annotated introns as pseudogene insertions (Figure 3D). We find 577 pseudogene insertions in the Hartwig cohort, exclusively in samples with somatically activated LINE mechanisms and enriched in the samples with the most deregulated LINE activity (Figure 3E).

#### Complex events

LINX classifies any cluster that has three or more junctions and is not resolved as a LINE source element as “complex.” Previous tools, notably ChainFinder,^4^ have been developed to systematically search for complex rearrangement patterns in tumors. We compared LINX and ChainFinder across 1,479 Hartwig cohort samples and found that, while in 22% of cases, LINX and ChainFinder produced near-identical clusters, the majority of junctions clustered by LINX are left unclustered by ChainFinder, while few SVs were exclusively clustered by ChainFinder (Figures S6A and S6B). We found this to be because of two main reasons: first, ChainFinder fails to cluster a large number of junctions that are highly proximate (<5 kb between breakends; Figure S6C) and, second, LINX employs a variety of clustering techniques to link distant junctions on the same chromosome arm that are not captured by ChainFinder (Figure S6D). The additional variants clustered by LINX compared with ChainFinder share a strikingly similar length distribution to the variants clustered by both tools (Figure S6E), including deletions, duplications, and inversions with lengths greater than 1 Mb, which are not normally found in simple events.

Conversely, in a small proportion (1.8%) of cases, junctions are clustered by ChainFinder and not by LINX. Ninety-five percent of these are deletions and tandem duplications <1 Mb in length that may also have occurred as independent events and be inadvertently clustered by ChainFinder (Figure S6E). In line with this hypothesis, we find that 20% of the deletions clustered by ChainFinder, but not by LINX, are in known fragile sites (Figure S6F) and often are phased in *trans*, suggesting that they likely occurred in different events.^12^

**Figure 4:**
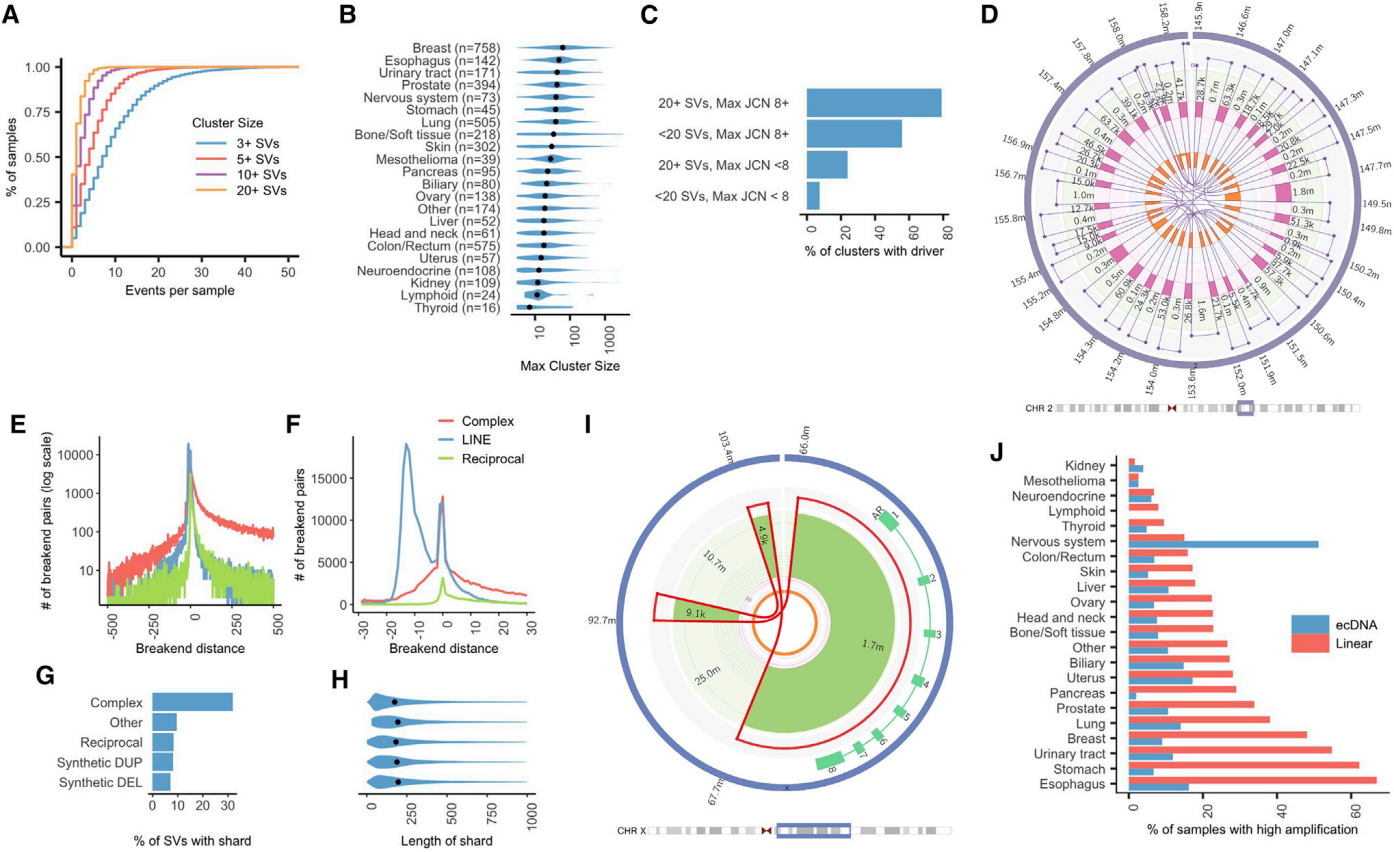
Complex rearrangements and high amplification (A) Cumulative distribution function plot of count of complex rearrangement clusters per sample with at least 3, 5, 10, and 20 variants. (B) Violin plot showing the distribution of the maximum number of variants in a single complex rearrangement cluster per sample, grouped by tumor type. Black dots indicate the median values for each tumor type. (C) Proportion of clusters contributing to at least one amplification, deletion, homozygous disruption, or LOH driver in a panel of cancer genes by complexity of cluster and maximum JCN. (D) Fully resolved chromothripsis event consisting of 31 structural variants affecting a 13-Mb region of chromosome 2 in HMF001571A, a prostate tumor. (E) Counts of occurrences of *trans*-phased breakends by distance between the breakends for complex events, LINE insertions, and two-break reciprocal clusters in the range of −500 to 500 bases (log scale). Negative distances indicate overlapping breakends and duplication at the rearrangement site. (F) Counts of occurrences of *trans*-phased breakends by distance between the breakends zoomed in to −30 to 30 bases. (G) Proportion of variants with at least one breakend joining a shard of less than 1 kb in length by resolved type for selected resolved types. (H) Violin plot showing the distribution of shard length by resolved type. (I) Double minute formed by three junctions in HMF003969A, a prostate tumor, and which amplifies known oncogene, *AR*, to a copy number of approximately 23. (J) Proportion of samples with ecDNA and linear amplifications by cancer type. See also Figures S6 and S7 and Table S5.

Across the Hartwig cohort, we found at least one complex event in 95% of tumors and at least one event of 20 or more junctions in 60% of tumors (Figure 4A). While there are relatively few complex events in any given tumor, they account for more than half of junctions overall. Complex clusters with >100 junctions were found in all cancer types, with breast cancer having the highest median maximum complex cluster size of 62 (Figure 4B). We observe that complex events with a higher number of junctions are more likely to disrupt or amplify a putative cancer driver gene. Overall, 12.7% of all complex clusters in the cohort contributed to a LOH, amplification, deletion, or disruption driver, but this rises to 39.1% for events with 20 or more junctions and 77% for events with more than 20 junctions and high amplification (junction copy number ≥8; Figure 4C).

LINX goes further than other clustering tools in that it allows not only for complex clusters to be identified but in many of cases is able to completely resolve such events into a consistent set of derivative chromosomes, including in chains with up to 33 junctions (Figure 4D). Uniquely, and critically for accurate chaining in these complex structures, LINX utilizes phased assembly output from GRIDSS to determine whether proximate facing breakends are *cis* or *trans* phased. We observe that *trans*-phased facing breakends, causing local duplication, are common in complex events and can often extend up to several hundred bases but only rarely extend beyond 30 bases in reciprocal events and mobile insertions (Figures 4E and 4F), suggesting a fundamentally different breaking mechanism in complex events, which may cause double-stranded breaks with hundreds of bases overlap. Proximate *cis*-phased breakends are even more common than *trans*-phased and resemble in length distribution the shards detected in simple events but with much higher frequency in complex clusters (Figures 4G and 4H). We frequently observe localized regions of scarring with multiple distinct shards sourced from the same location, sometimes with overlapping template sequences.

#### Amplification mechanisms

Regions of high amplification are among the most complex events in tumors, as they require iterative and repeated cycles of synthesis or unequal segregation to form. There are two well-known key distinct biological mechanisms that create highly amplified rearrangements: repeated cycles of breakage fusion bridge (BFB) and stochastic amplification of circular extrachromosomal DNA by asymmetric segregation during cell division (ecDNA). ecDNA (Figures 4I and S7A) may arise from any event that creates simultaneous multiple double-stranded breaks on the same chromosomal arm, with one or more chromosomal segments repairing to form a circular structure without a centromere. BFB (Figure S7B), on the other hand, is triggered by the formation via translocation or foldback inversion of a chromosome with two centromeres, arising from either multiple concurrent double-stranded breaks or telomere erosion, and leads to duplication of chromosomal segments within a linear chromosome.

Despite these significant differences in mechanism, distinguishing between ecDNA and BFB is non-trivial based on short-read sequencing data but is essential in order to understand the diversity of amplification drivers in tumors and may be relevant to the prognosis or treatment of certain tumors.^23^ The key difficulties in discrimination are that both mechanisms can leave a similar footprint, as both may arise out of complex shattering events and are highly shaped by the same selection processes, both positive (amplification of key oncogenes) and negative (constraints on amplifications of other proximate genes).

LINX employs a set of heuristics to identify subsets of clusters as likely ecDNA. The key principle used to identify ecDNA is to look for high junction copy number (JCN) structural variants adjacent to low-copy-number regions that can be chained into a closed or predominantly closed loop. LINX also checks that the high JCN cannot be explained by compounding linear amplification mechanisms, by comparing the JCN of the candidate ecDNA junctions with the maximal amplification impact of foldback inversions (hallmarks of BFB) and other junctions that link closed segments of the ecDNA to other regions of the genome (Methods S1). To validate the ecDNA heuristic, we ran LINX on a set of 13 WGS neurosphere-cultured glioblastoma samples that had been previously analyzed^24^ for ecDNA with Amplicon Architect.^6^ LINX and Amplicon Architect called ecDNA for an identical set of 19 oncogenes across the 13 samples (Table S5), including the 11 samples that were orthogonally validated by fluorescence *in situ* hybridization (FISH).

Applying the heuristic to the Hartwig cohort, we found ecDNA to be a relatively uncommon event present in 9.9% of all tumors, with the highest frequency in CNS tumors (51%; Figure 4J). This is lower than found in a large recent pan-cancer cohort analysis of WGS using AmpliconArchitect,^23^ which found a pan-cancer prevalence of 14%. We observe that, overall, 12% of putative amplification drivers identified in the Hartwig cohort are associated with ecDNA events (Figure S7C) but that this rate increases for more highly amplified events to greater than 40% for events with maximum JCN > 32. The relative rate of ecDNA is the highest for *EGFR* (Figure S7D), but this appears to be highly specific to CNS tumors (where 87% of *EGFR* amplifications are associated with ecDNA), whereas for lung tumors (where epidermal growth factor receptor [EGFR] amplification is also common) and other cancer types, the rates of ecDNA are only 11% and 21%, respectively, similar to that of other well-known oncogenes (Figure S7E).

The high-amplification events that do not meet the ecDNA criteria are assumed to be formed via linear amplification. While we find that 76% of these events have at least one foldback inversion, suggesting a BFB process, in many events, the foldback JCN cannot explain the full amplification, and in the remaining events, LINX identifies no foldback events at all (Figure S7F). The majority of these are unlikely to be ecDNA, however, because there is no obvious set of junctions and segments that can be closed into a circle with a consistent copy number. Some events, such as the exceptionally complex amplifications of *MDM2* and *CDK4* common in liposarcoma,^3^ may not fall neatly into either an ecDNA or BFB classification (Figure S7G) and have recently been proposed to be a novel rearrangement class termed “tyfonas.”^12^

#### Detection of clinically relevant pathogenic rearrangements

**Figure 5:**
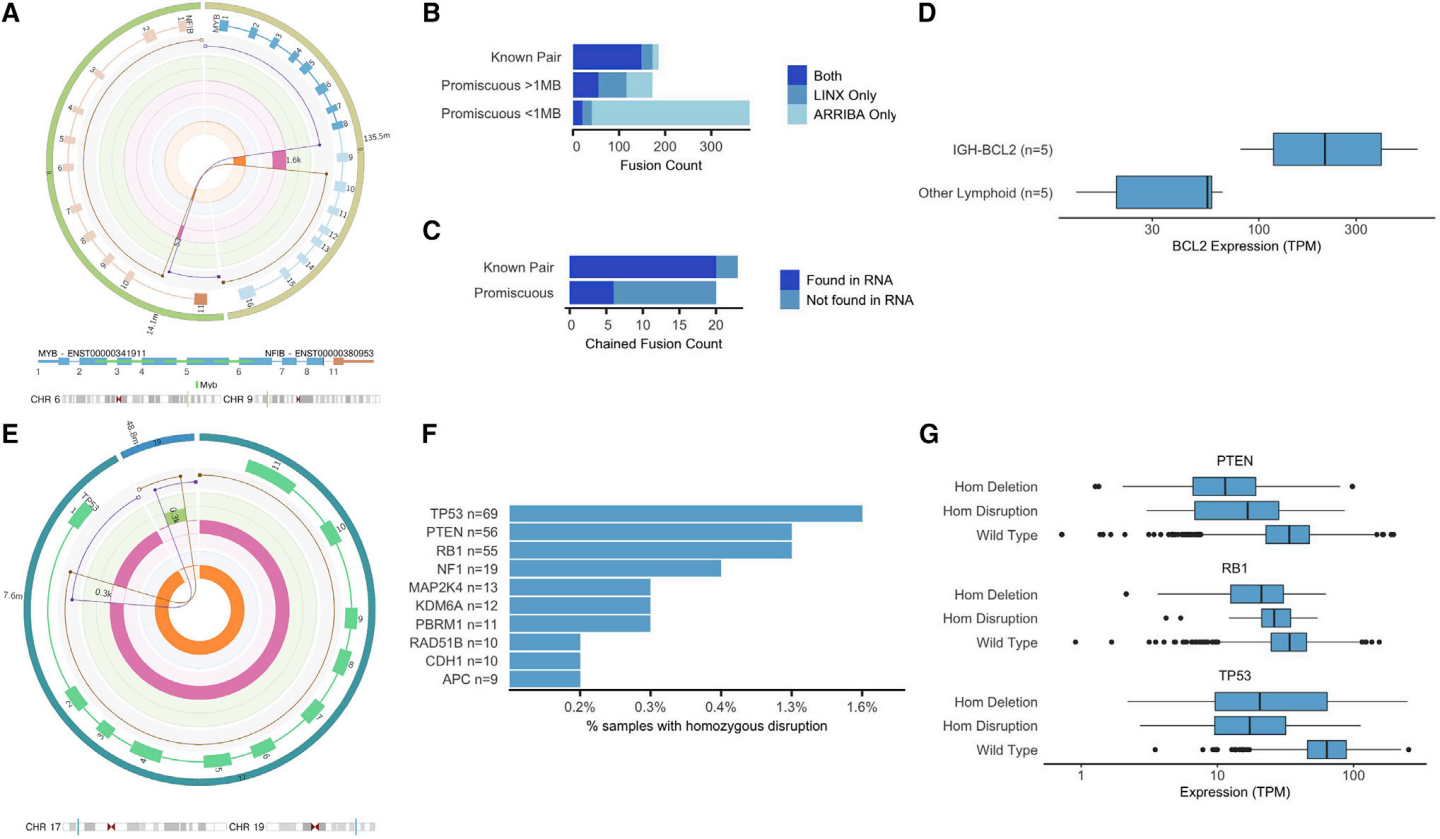
Clinically relevant rearrangements (A) A *MYB*-*NFIB* fusion caused by a reciprocal translocation in HMF000780A, a salivary gland tumor. The translocation links exons 1–8 in *MYB* to exon 11 in *NFIB*. (B) Comparison of LINX fusion predictions in Hartwig cohort to Arriba fusion predictions from orthogonal RNA sequencing for known pairs and promiscuous fusion partners. Promiscuous fusions of less than 1 Mb length are shown separately, as they may occur from readthrough transcripts and not be associated with a genomic rearrangement. (C) Count of LINX chained fusion predictions for known and promiscuous fusion and whether they are also found to be expressed in RNA by Arriba. (D) Distribution of *BCL2* expression in lymphoid samples with and without a predicted pathogenic *IGH-BCL2* rearrangement. Box: 25^th^–75^th^ percentile; whiskers: data within 1.5 times the interquartile range (IQR). (E) Reciprocal translocation affecting *TP53* in HMF001913A, a prostate tumor. The two predicted derivative chromosomes overlap by approximately 300 bases on both ends but are *trans* phased, which rules out the possibility of a templated insertion at either location. Although the *TP53* copy number alternates between one and two, no derivative chromosome contains the full gene and the gene is homozygously disrupted. (F) Prevalence of homozygous disruption drivers for top 10 most affected tumor-suppressor genes. (G) Distribution of gene expression in Hartwig cohort for samples with homozygous deletion, homozygous disruption, and wild type for each of *RB1*, *TP53*, and *PTEN*. box: 25^th^–75^th^ percentile; whiskers: data within 1.5 times the IQR. See also Figures S8 and S9 and Tables S6, S7, S8, and S9.

LINX calls a diverse and comprehensive range of fusions and pathogenic rearrangements (Figures 5A and S8A–S8D). We orthogonally validated LINX’s pathogenic fusion predictions by comparing them with fusions predicted from RNA sequencing (RNA-seq) data taken from the same samples. For the RNA comparison, we used Arriba, one of the best performing RNA fusion callers,^25^ using a curated list of 391 known pathogenic fusion pairs (Table S6). Across 1,924 Hartwig cohort samples with matched RNA, 148/173 in-frame fusions (86%) predicted by LINX were also found by Arriba (Figure 5B; Table S7). Of the 25 fusions not identified in RNA, 13 matched the characteristic tumor type of the known fusion pair (nine of which were *TMPRSS2-ERG* fusions in prostate cancer) and are likely to be pathogenic but with insufficient expression to be detected in the RNA. A further two cases predicted by LINX were found by Arriba but only in out-of-frame transcripts. Thirteen known pair fusions were predicted by Arriba, but not by LINX, seven of which involve gene pairs less than one million bases apart on the same chromosome and may be caused by readthrough transcripts^26^ or circularized RNA^27^ unrelated to structural rearrangements in the DNA.

In addition to known pathogenic fusion pairs, 63 cancer-related fusion genes were curated as promiscuous 5′ and 3ʹ fusion partners. Among these, LINX identified a further 152 candidate in-frame fusions, 74 (49%) of which were also detected in RNA. Arriba detected 397 additional promiscuous candidates, but 86% of these were proximate on the same chromosome and are likely readthrough transcripts with no genomic rearrangement. Altogether, 43 of the 325 (13%) known and promiscuous fusion predictions were chained fusions involving multiple junctions, 26 (60%) of which were validated in the RNA-seq data (Figure 5C), highlighting the utility of chaining of derivative chromosomes for DNA fusion calling. TMPRSS2-ERG was the only fusion that LINX found to be recurrently chained in the cohort, accounting for 14 of the 43 predicted chained fusions, all in prostate cancers.

Immunoglobulin enhancer rearrangements are a distinct class of pathogenic rearrangements, common in B cell tumors where errors in VDJ recombination and/or isoform switching in the *IGH*, *IGK*, and *IGL* regions may lead to pathogenic rearrangements, driving high expression of known oncogenes through regulatory element repositioning.^28^ Although these typically do not make a novel protein fusion product, LINX predicts these pathogenic rearrangements based on the breakend in the *IGH*, *IGK*, and *IGL* regions with orientation and position matching locations commonly observed in B cell tumors.^28^ Among 10 lymphoid samples with matching RNA in the cohort, LINX found six such rearrangements, including five cases of *IGH-BCL2* and one case of *IGH-MYC*. The five identified samples with *IGH-BCL2* rearrangements have significantly higher expression (p = 0.008) of *BCL2* than the five lymphoid samples with no *BCL2* rearrangement detected (Figure 5D).

LINX also identifies disruptive intragenic rearrangements that may cause exonic deletions and duplications. Our knowledge base includes nine such rearrangements known to be pathogenic and two that we have deemed likely pathogenic due to high recurrence in the Hartwig cohort. Three of the known pathogenic exon rearrangements were detected by LINX in at least five samples with paired RNA in our cohort: *EGFRvII* (n = 6), *EGFRvIII* (n = 14), and *CTNNB1* exon three deletion (n = 6). In all cases with an event detected by LINX in the DNA, we found RNA fragments that supported novel splice junctions in the matched RNA (Figure S8E). Only one other sample in the complete cohort (n = 1,924) had more than one fragment supporting any of these alternative splice junctions (a gastrointestinal stromal tumor with three fragments supporting EGFRvII but with no evidence of rearrangement in EGFR), suggesting a low false-negative rate in LINX.

In addition to producing novel oncogenic proteins and over-expression of well-known oncogenes, rearrangements may also lead to tumorigenesis by disrupting the function of tumor-suppressor genes. To capture this, LINX annotates every breakend that overlaps a gene, determines whether it is disruptive to the gene, and reports the number of undisrupted copies. In cases of reciprocal translocations (Figure 5E), reciprocal inversions (Figure S9A), complex break events (Figure S9B), or tandem duplications that overlap at least one exon (Figure S9C), a gene may be disrupted on all remaining copies, even though the copy number is greater than zero for all exonic bases.^29^ We term this type of genomic rearrangement a “homozygous disruption.” Homozygous disruptions cannot readily be detected by standard panel or whole-exome sequencing, since intronic sequences are typically not included in such panels and they are copy neutral in exonic regions.

We find homozygous disruptions to be a common driver in the Hartwig cohort, with 9% of samples containing at least one homozygous disruption in a panel of 448 curated cancer-related genes (Table S8). Three well-known tumor-suppressor genes had homozygous disruptions in more than 1% of the cohort: *TP53* (n = 69), *PTEN* (n = 56), and *RB1* (n = 55; Figure 5F). Supporting the functional impact of these events, we found significantly lower expression for each of these genes (TP53: p = 2 × 10^−16^; PTEN: p = 2 × 10^−6^; RB1: p = 2 × 10^−3^) in samples with predicted homozygous disruptions compared with samples with at least one intact copy (Figure 5G) and similar mean fold change in expression compared with samples with homozygous deletions (TP53: 0.30 versus 0.40; PTEN: 0.47 versus 0.37; RB1: 0.68 versus 0.60 for disruptions and deletions, respectively). We also performed a genome-wide search for genes with enrichment of homozygous disruptions and found 35 significantly enriched genes, including 16 well-known tumor suppressors, 14 genes immediately adjacent to tumor-suppressor genes, and three highly recurrent oncogenic fusion partners (Table S9). Intriguingly, we found an additional two genes also enriched in homozygous disruptions, but not widely characterized as tumor-suppressor genes: *PSIP1* (five observations; q = 0.006), which has previously also shown to be significantly enriched in truncating point mutations,^30^ and *USP43* (six observations; q = 0.01), a recently proposed tumor suppressor.^31^

#### Visualization

**Figure 6:**
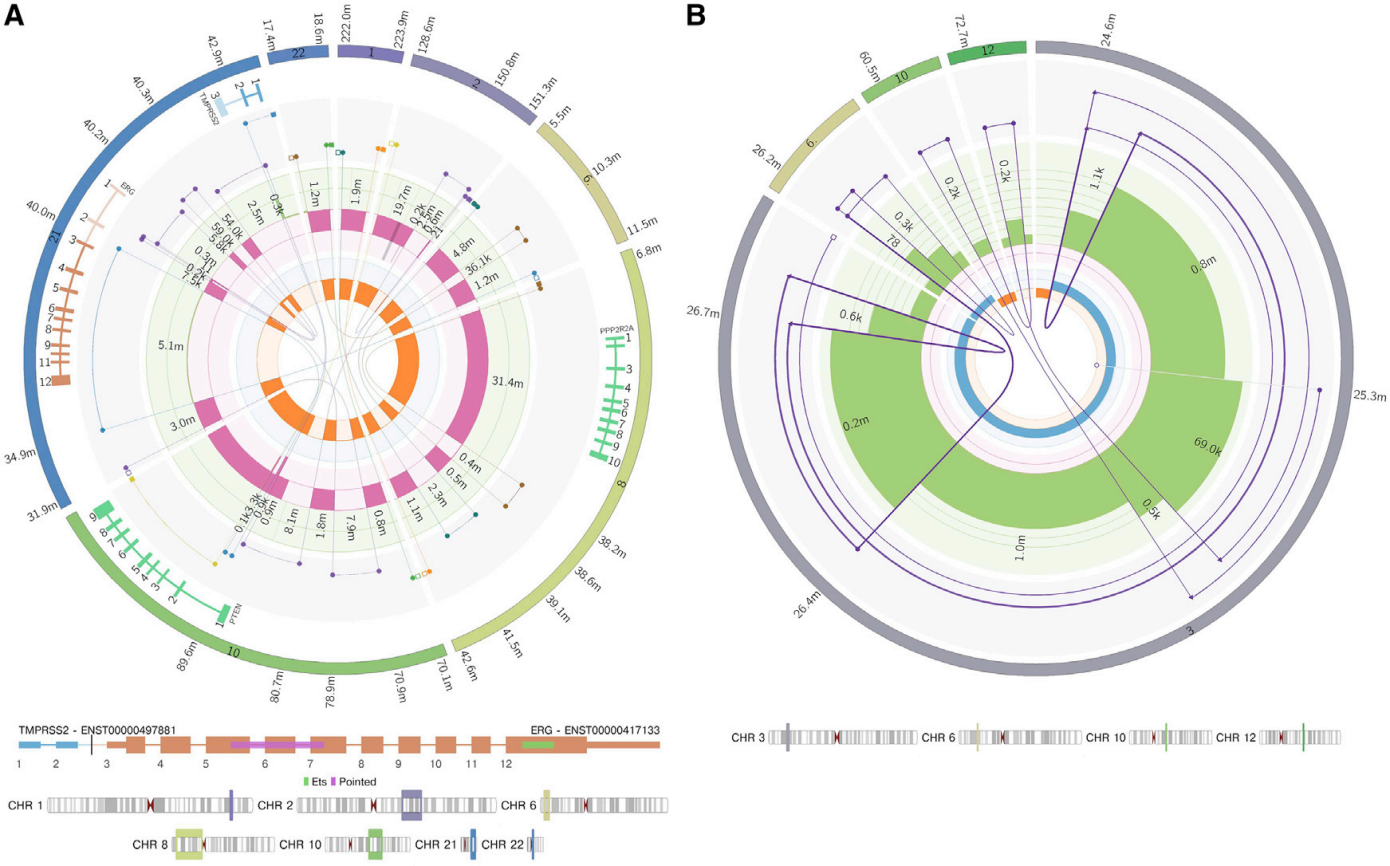
Complex event visualization (A) Chromoplexy-like cluster formed from 19 break junctions across seven chromosomes in HMF001596B, a prostate tumor. The rearrangement leads to three distinct putative drivers in a single event, including a chained *TMPRSS2-ERG* fusion with two hops; a loss of heterozygosity for *PPP2R2A*, which also has a stop-gained point mutation (not shown); and an intronic homozygous disruption of *PTEN*. (B) Breakage fusion bridge event affecting the P arm of chromosome 3 in the melanoma cell line COLO829T. The predicted derivative chromosome has a copy number of two and can be traced outwards starting from the centromere on chromosome 3, traversing two simple foldbacks and two chained foldbacks and finishing on a single breakend at chr3:25.3M, which from the insert sequence can be inferred to be connected to a centromeric satellite region (likely chromosome 1, which has a copy number gain of two over the centromere from P to Q arm and which appears to be connected to chromosome 3 in unpublished SKY karyotype figures; http://www.pawefish.path.cam.ac.uk/OtherCellLineDescriptions/COLO829.html). One chained foldback at chr3:26.4M includes a genomic shard from chr6 of approximately 400 bases, which has itself been replicated and internally disrupted by the foldback event. The other chained foldback at chr3:25.4M includes two consecutive genomic shards inserted from chromosome 10 and 12 of approximately 200 bases each.

LINX produces detailed visualizations of the rearrangements in the tumor genome that allow further insights into complex rearrangements. LINX supports either drawing all rearrangements in a cluster or all the rearrangements on a chromosome, creating an integrated Circos output^32^ showing copy number changes, clustered SVs, the derivative chromosome predictions, and impacted genes, including protein domain annotation for gene fusions, all on the same diagram. The visualizations use a log-based position scaling between events so that small- and large-scale structures can both be inspected on a single chart. Combined with the circular representation, these features allow unprecedented resolution of complex structures across a broad array of event types, including chromoplexy (Figure 6A) and complex BFB amplification events (Figure 6B). Methods S1 includes a walkthrough and explanation of all LINX figures, covering the complete SV landscape of the COLO829T melanoma cancer cell line, which has been proposed as a somatic reference standard for cancer-genome sequencing.^33^^,^^34^

##### Evaluation on an independent cohort

To assess broader utility of the tool set and the reproducibility of our results, we compared the findings on the Hartwig cohort with a subset of 1,541 samples from the independently sequenced PCAWG pan-cancer cohort (Table S10).^35^ The PCAWG samples analyzed also cover a diverse range of tumor types but, unlike the Hartwig cohort, contain almost exclusively primary cancer samples and are sequenced to a lower average coverage of depth (38×–60× PCAWG compared with median 106× for HMF).

We observed largely the same structural variant patterns across the two cohorts (Figure S10A). The length distributions of deletions, duplications, and inversions were highly similar for both simple and complex events across the two cohorts (Figure S10B). We also observed a very similar preponderance and length distribution of genomic shards across all event types (Figure S10C). Furthermore, we found that the length distributions of the synthetic events in the PCAWG cohort closely replicated the results found in the Hartwig cohort (Figure S10D). Likewise, the reciprocal duplication events we identified in the Hartwig cohort were also present in PCAWG, with the same length patterns of tandem duplication signatures for samples with *BRCA1*, *CDK12*, and *CCNE1* drivers (Figure S10E). Driver-related rearrangement patterns were also similar between the PCAWG and Hartwig cohorts. While the overall rates of samples with high-amplification events were lower in the primary cancers (22% PCAWG; 41% HMF), the proportion accounted for by ecDNA was similar (28% PCAWG; 24% HMF; Figure S10F). We also found homozygous disruptions events impacting tumor-suppressor genes (TSGs) in the PCAWG cohort. Indeed, the top four driver genes with putative homozygous disruption drivers were the same in both datasets (Figure S10G).

Overall, the high reproducibility of these results in the independently sequenced PCAWG cohort lends weight both to the utility of LINX and the universality of the observed patterns across both metastatic and primary cancers.

## Discussion

We have shown that LINX can help understand highly rearranged cancer genomes in multiple ways. Other recent publications on complex somatic rearrangements^1^^,^^12^ have developed tools, such as ClusterSV and JaBbA, that have significant feature overlap with LINX. Each of these approaches also utilizes a base-pair-consistent SV/CN call set to cluster SVs, classify certain types of rearrangements, and assess downstream impact.

However, LINX differs from existing approaches in several key aspects. First, LINX clusters use copy number consistency constraints in addition to SV proximity. Second, LINX chains SVs to reconstruct the derivative chromosomes caused by each rearrangement event, including partial reconstruction for incomplete events. Third, LINX performs comprehensive classification. Every SV is classified, including mobile element translocations. Fourth, LINX utilizes single-breakend SV calls. The single-breakend repeat annotations provided by GRIDSS enable LINX to classify mobile element translocations as well as cluster complex events overlapping centromeric repeats. Fifth, LINX’s rearrangement model allows for genomic shards to be inserted in any event type. The size distribution of sharded events indicates this approach is sound, at least for simple events, and this approach considerably simplifies the classification scheme. Sixth, LINX utilizes a nonlinear Circos-style visualization format that enables even quite complex rearrangements to be visually interpretable. Finally, LINX provides the most comprehensive genomic rearrangement functional impact analysis currently available. To the best of our knowledge, LINX is the only tool that reports homozygous disruptions and the only tool that can identify chained fusions from DNA-seq data alone, both of which can lead to clinically relevant rearrangements in tumors.

The challenges in understanding the complexity of rearrangements in tumor genomes can be daunting. The diversity of overlapping or converging biological mechanisms that may cause similar rearrangement patterns means that it may be perilous to analyze any one rearrangement as a standalone analysis. By exhaustively classifying all rearrangements, LINX is a robust foundation for more detailed analysis of specific rearrangement patterns, including structural variant signatures, complex shattering events, and high-amplification drivers as well as dissection of the underlying molecular mechanisms, DNA replication, and repair components involved. The full LINX analysis results on the Hartwig cohort are available via data request and can be paired with clinical data and other whole-genome analyses for further in-depth research.

WGS offers the promise of a single comprehensive test for all genomic alterations for both routine diagnostics and future biomarker discovery. LINX takes a step toward that goal by both comprehensively calling clinically relevant fusions from DNA with similar precision and sensitivity to gold standard RNA-seq methods and by identifying homozygous disruptions, an important class of drivers of tumorigenesis that cannot readily be detected by standard-of-care methods.

### Limitations of the study

There are many potential sources of error that can confound correct interpretation of complex genomic rearrangements, including sample preparation, sequencing errors and coverage biases (such as GC bias), inaccurate fitting of sample purity and ploidy, false-positive or false-negative structural variant calls, and inaccurate local copy number measurement. Depth of coverage and sequencing quality are important considerations here. While we have shown that LINX can find highly similar results on the PCAWG dataset, which has, on average, half the sequencing coverage of the Hartwig cohort, lower depth coverage and/or lower quality sequencing is associated with higher false-negative rates of structural variants^9^ and will result in less complete reconstructions.

Furthermore, while LINX has been optimized for short-read technology, the short-read length is ultimately the key limitation in interpretation, because it limits the phasing of proximate variants and accurate identification of events in long repetitive regions. Nevertheless, in practice, LINX is able to resolve many structures via various chaining and clustering heuristics, but for more complex events, particularly highly rearranged focal regions, errors are inevitable and the chaining is only partial and representative. While we have performed extensive comparison of LINX against other tools and can validate some of LINX’s chaining predictions orthogonally via RNA evidence for chained fusions, there are, as yet, no representative tumor genomes with a fully resolved chromosomal structure for comparison as a truth set. Long-read sequencing technologies^36^ can phase more distant breakpoints and are likely better suited for resolving complex events, although those technologies typically perform less well for small variant detection. Pairing short- and long-read technologies will no doubt lead to further advances in our understanding of the mechanisms and role of genomic rearrangements in tumorigenesis.

## STAR★Methods

### Key resources table

**Table:** 

REAGENT or RESOURCE	SOURCE	IDENTIFIER
**Deposited data**		

HMF WGS/WTS BAMs	Priestley et al., 2019^8^	https://www.hartwigmedicalfoundation.nl/en/database/
PCAWG WGS BAMs	Consortium and The ICGC/TCGA Pan-Cancer Analysis of Whole Genomes Consortium, 2020^35^	http://dcc.icgc.org/pcawg/
WGS glioblastoma neurosphere cultures BAMs	deCarvalho et al., 2018^24^	EGA accession: EGAS00001001878

**Software and algorithms**		

LINX v1.12	This paper	https://github.com/hartwigmedical/hmftools/tree/master/linx
GRIDSS2 v2.9.3	Cameron et al., 2021^9^	https://github.com/PapenfussLab/gridss
PURPLE v2.48	Priestley et al., 2019^8^	https://github.com/hartwigmedical/hmftools/tree/master/purple
STAR 2.7.3a	Dobin et al., 2013^37^	https://github.com/alexdobin/STAR
Isofox v1.0	Hartwig Medical Foundation	https://github.com/hartwigmedical/hmftools/tree/master/isofox
ChainFinder v1.0.1	Baca et al., 2013^4^	https://software.broadinstitute.org/cancer/cga/chainfinder
Circos	Krzywinski et al. 2009^32^	http://circos.ca/

### Resource availability

#### Lead contact

Further information and requests for resources and reagents should be directed to and will be fulfilled by the lead contact, Peter Priestley (p.priestley@hartwigmedicalfoundation.nl).

#### Materials availability

This study did not generate any new reagents.

### Experimental model and subject details

The patient cohort was derived from the Hartwig Medical Foundation Cohort for which the sample collection and whole genome sequencing and alignment to the GRCH37 reference genome has previously been described.^8^ We filtered for the highest purity sample from each patient from tumor samples with purity ≥ 20% and with no QC warnings or failures, yielding 4,378 paired tumor-normal whole genome samples in total. An additional 1,774 paired tumor-normal sample BAMS were obtained from PCAWG, 1,541 of which passed QC warnings and purity filters. For 1,924 HMF samples paired whole transcriptome sequence data were also analyzed.

### Method details

#### Analysis of structural variation and copy number alterations

GRIDSS^9^ v2.93 and PURPLE^8^ v2.48 were used for copy number and structural variant inputs for LINX. LINX v1.12 was used for all analyses in this paper and is described in detail in Methods S1.

#### RNA validation

The RNA-seq was aligned to the GRCH37 genome using STAR 2.7.3a.^37^ Gene expression was calculated using Isofox v1.0, which uses an expectation maximisation algorithm to estimate transcript abundance from genome aligned RNA-seq data, with default parameters. Isofox was also used to count the RNA fragments supporting novel splice junctions predicted in LINX for exon deletions and duplications. Isofox is described in detail at https://github.com/hartwigmedical/hmftools/tree/master/isofox.

Known pathogenic pair and promiscuous gene fusions predictions in the DNA were compared to passing fusion calls in the RNA by Arriba (https://github.com/suhrig/arriba). Fusions were considered to be matched if the gene pair matched between RNA and DNA. Mean TPM fold change was calculated as 2 to the power of the difference in mean(log2(TPM)) between groups of samples.

#### Complex event validation

We compared LINX to ChainFinder^4^ v1.0.1 on 2,840 samples from the Hartwig cohort. ChainFinder was run with default parameters. Both LINX and ChainFinder were run using the same GRIDSS/PURPLE input data. Only 1,479 samples for which ChainFinder completed within 24 hours were included in the comparison. ChainFinder clusters of 3 or more variants were considered equivalent to LINX’s COMPLEX classification. For each individual variant we determined whether it was clustered in LINX, in ChainFinder or in both as well as the size of the cluster in each tool.

#### LINE insertion validation

We ran LINX on 75 WGS samples from the PCAWG cohort (Table S2) which had previously been run with TraFiC-mem.^5^ Insertions were considered matched between the tools if the predicted insertion site was within 50 bases.

#### ecDNA validation

We ran LINX on 13 previously analysed^24^ WGS glioblastoma neurosphere cultures sequenced to ∼10x depth and compared the ecDNA predictions of Linx to those of the AmpliconArchitect tool and FISH. We matched the ecDNA predictions by amplified oncogene per sample.

#### Genes enriched in homozygous disruptions

We estimated a background rate of homozygous disruptions by dividing the total number of observed homozygous disruptions across the full Hartwig cohort by the total length of all annotated genes in the Hartwig cohort. For each gene we then compared the observed number of homozygous distribution to the expected number taking into account the global rate and the length of the specific genes using a Poisson distribution and correcting for false discovery. Genes with a false discovery rate of less than 0.1 were reported.

### Quantification and statistical analysis

Statistical tests are described in figure legends. All significance values presented for comparisons of both gene expression and counts of rearrangement types are calculated using a two-tailed Mann–Whitney U-test.

## Data Availability

All raw (BAM), analysed (VCF, SV, purity copy number data) germline, and somatic genomic data and LINX results from the Hartwig cohort were obtained from the Hartwig Medical Foundation (Data request DR-005). Standardized procedures and request forms for access to this data, including LINX analysis results, can be found at https://www.hartwigmedicalfoundation.nl/en. LINX is freely available as open source software from the Hartwig Medical Foundation (https://github.com/hartwigmedical/hmftools/tree/master/linx) under a GPLv3 license. Reference data required to run LINX on hg19 or hg38 is available from https://resources.hartwigmedicalfoundation.nl. LINX can be run from raw paired tumor-normal FASTQ files as part of Hartwig’s open source cloud-based cancer analysis pipeline (https://github.com/hartwigmedical/platinum). Alternatively, a docker image is available from dockerhub as gridss/gridss-purple-linx to run GRIDSS, PURPLE, and LINX together from tumor and normal BAMs.

## Supplemental information

Document S1. Figures S1–S10 and Methods S1

Table S1. Panel of driver genes used in study, related to STAR Methods

Table S2. Hartwig DNA and RNA samples used in study, related to STAR Methods

Table S3. Counts of structural variants by sample and classification, related to Figure 2

Table S4. LINX and Traffic-Mem LINE insertion predictions for 75 PCAWG samples, related to Figure 3

Table S5. ecDNA validation on 13 glioblastoma samples, related to Figure 4

Table S6. Curated fusion knowledge base used to search for pathogenic rearrangements, related to Figure 5

Table S7. In-frame pathogenic rearrangements detected in 1,924 samples by DNA and/or RNA fusion calling, related to Figure 5

Table S8. Pathogenic homozygous disruptions detected in tumor-suppressor genes in 4,358 samples, related to Figure 5

Table S9. Genes enriched in homozygous disruptions, related to Figure 5

Table S10. PCAWG samples used in this study, related to STAR Methods

Document S2. Article plus supplemental information
